# Supplementation of a lacto-fermented rapeseed-seaweed blend promotes gut microbial- and gut immune-modulation in weaner piglets

**DOI:** 10.1186/s40104-021-00601-2

**Published:** 2021-07-20

**Authors:** Yan Hui, Paulina Tamez-Hidalgo, Tomasz Cieplak, Gizaw Dabessa Satessa, Witold Kot, Søren Kjærulff, Mette Olaf Nielsen, Dennis Sandris Nielsen, Lukasz Krych

**Affiliations:** 1 Department of Food Science, Faculty of Science, University of Copenhagen, Rolighedsvej 26, DK-1958 Frederiksberg C, Denmark; 2 Fermentationexperts A/S. Vorbassevej 12, DK-6622 Bække, Denmark; 3 Department of Veterinary and Animal Sciences, Faculty of Health and Medical Sciences, University of Copenhagen, Grønnegårdsvej 3, DK-1870 Frederiksberg C, Denmark; 4 Department of Plant and Environmental Sciences, Faculty of Science, University of Copenhagen, Rolighedsvej 26, DK-1958 Frederiksberg C, Denmark; 5 Department of Animal Science, Faculty of Technical Sciences, Aarhus University, Blichers Allé 20, DK-8830 Tjele, Denmark

**Keywords:** Amplicon sequencing, Colon microbiota, Fermented feed, Gut barrier

## Abstract

**Background:**

The direct use of medical zinc oxide in feed will be abandoned after 2022 in Europe, leaving an urgent need for substitutes to prevent post-weaning disorders.

**Results:**

This study investigated the effect of using rapeseed-seaweed blend (rapeseed meal added two brown macroalgae species *Ascophylum nodosum* and *Saccharina latissima*) fermented by lactobacilli (FRS) as feed ingredients in piglet weaning. From d 28 of life to d 85, the piglets were fed one of three different feeding regimens (*n* = 230 each) with inclusion of 0%, 2.5% and 5% FRS. In this period, no significant difference of piglet performance was found among the three groups. From a subset of piglets (*n* = 10 from each treatment), blood samples for hematology, biochemistry and immunoglobulin analysis, colon digesta for microbiome analysis, and jejunum and colon tissues for histopathological analyses were collected. The piglets fed with 2.5% FRS manifested alleviated intraepithelial and stromal lymphocytes infiltration in the gut, enhanced colon mucosa barrier relative to the 0% FRS group. The colon microbiota composition was determined using V3 and V1-V8 region 16S rRNA gene amplicon sequencing by Illumina NextSeq and Oxford Nanopore MinION, respectively. The two amplicon sequencing strategies showed high consistency between the detected bacteria. Both sequencing strategies indicated that inclusion of FRS reshaped the colon microbiome of weaned piglets with increased Shannon diversity. *Prevotella stercorea* was verified by both methods to be more abundant in the piglets supplied with FRS feed, and its abundance was positively correlated with colonic mucosa thickness but negatively correlated with blood concentrations of leucocytes and IgG.

**Conclusions:**

FRS supplementation relieved the gut lymphocyte infiltration of the weaned piglets, improved the colon mucosa barrier with altered microbiota composition. Increasing the dietary inclusion of FRS from 2.5% to 5% did not lead to further improvements.

**Supplementary Information:**

The online version contains supplementary material available at 10.1186/s40104-021-00601-2.

## Background

A healthy hindgut is essential for the optimal nutrient utilization and host health. Essentially, the gut microbes ferment and digest macronutrients such as fibers in the colon [1, 2]. Microbial metabolism benefits the colonic cells and immune system by providing short-chain fatty acids (SCFAs) like butyrate and acetate [1]. Microbial colonization brings benefits for the host but also potential threats e.g., inflammatory responses by infections. Thus, microbial communication with the immune system balances on a thin line to preserve homeostasis [3] of what to attack and what to tolerate [4].

In pig production, the weaning period is characterized by a change in diet from milk to solid feed, separation from the mother and aggregation in a pen with piglets from other litters. It is a stressful period in pig life and has a high risk of morbidities such as diarrhea. In-feed zinc oxide used to be a prevalent choice for prophylaxis, but according to the European Union regulations, zinc oxide shall no longer be directly used in feed or water from June 2022 [5]. This leaves an urgent need for new prophylactic substitutes. Inclusion of pre-fermented feed is a promising strategy to ameliorate the post-weaning disorders for its effective act to improve gastrointestinal health and enhance livestock performance in production [6–8]. The process of microbial fermentation degrades antinutritional compounds and macronutrients in feed, increasing the nutrient bioavailability and nutritional value [9, 10]. Besides, the microorganisms in fermented feed have been proposed to inhibit the overgrowth of opportunistic pathogens, sustain gut microbiome homeostasis [11] and boost host immune system [12]. A meta-analysis has shown that fermented feed can increase the growth and performance of both weaner and growing pigs [13].

Rapeseed meal is a by-product after the oil has been extracted and is generally used as a protein source in animal diets [14]. However, it has a lower protein digestibility as compared to soybean meal. Brown seaweed is acknowledged as a reliable source of health-promoting phytochemicals [15] like laminarin [16]. The phlorotannin-rich extract from brown seaweed *Ascophyllum nodosum* has been reported to show anti-inflammatory benefits [17] and *Saccharina latissima* is identified as the producers of the antimicrobial compound 2,4-diacetylphloroglucinol [18]. However, the poor digestibility affected their wide use in feed. Lacto-fermentation can hydrolyze protein and fibers down to a more soluble matrix, and reduce the naturally present antinutritional factors. We have previously reported that dietary supplementation with rapeseed-seaweed blend (rapeseed meal added two brown macroalgae species *Saccharina latissima* and *Ascophylum nodosum*) fermented by lactobacilli (FRS) could modulate the gut barrier function of piglets and increase the production performance [5].

In the present study, we tested whether inclusion of FRS to weaner diets could affect the gut microbiome and the piglet production performance and influence interactions between the diet-induced gut microbiome modulation and host health in the weaning period. To trace the gut microbiome shifts, we profiled the piglet colon content using the V3 and V1-V8 region 16S rRNA gene amplicon sequencing by second (Illumina, NextSeq) and third generation (Oxford Nanopore Technologies, MinION) sequencing platform, respectively.

## Methods

### Preparation of fermented rapeseed-seaweed feed

The FRS feed provided by FermentationExperts (Denmark) was a blend of rapeseed meal (*Brassica napus*), wheat bran (*Triticum eastivum*) and two types of brown seaweed (*Saccharina latissima* and *Ascophylum nodosum*) prepared via a controlled two-step solid state fermentation. The inoculum consisted of three lactic acid bacteria: *Pediococcus acidilactici* (DSM 16243), *Pediococcus pentosaceus* (DSM 12834) and *Lactobacillus plantarum* (DSM 12837). The addition of the inoculant controlled the process by acidifying the blend within the first 24 h to assure an almost entirely anaerobic process. The process continued for 11 d at 38 °C. The fermented material was then dried in a spin flash dryer, with a temperature setting (in a range of 40 to 75 °C) and pass-through-speed that preserved the viable bacteria and the microbial thermolabile metabolites (patent no. WO2013029632A1).

### Animal feeding and performance recording

The feeding trial was carried out on a commercial pig farm (Kawiks Farm, Patoki 23. 98–170 Widawa. Province. Lodz city, Poland) in 2018. The trial procedure and sample collection were approved by the Local Ethical Commission of Olsztyn University of Life Sciences (Olsztyn, Poland) with regards to experimentation and animal care. A total of 690 piglets were tested under three different feeding regimens (230 piglets per feeding treatment) from 28 days of age (10 d before weaning) until 85 days of age when the piglets exited the nursing unit. One group was a control group fed a basal feed according to Danish nutritional recommendations [19] (0% FRS), and the other two groups received supplementation of 2.5% or 5% FRS to the basal feed. In this period, piglets were first supplied with three-week pre-starter diet, followed by five-week starter diet. All diets were subjected to standard analyses of pig feed in Denmark and Poland, where the metabolizable energy contents were determined through laboratory analyses of chemical composition, total enzyme digestible organic matter and enzyme digestible organic matter at ileum. These analyses were carried out in a commercial and certified feed factory Wola-Pasze Sp. Z o.o. (Biała Podlaska, Poland). Vitamin E was added to follow the standard feed formulation on the farm. None of the diets included growth promoters, prescription antibiotics or zinc oxide. Table 1 summarized the ingredients and nutritional values of feed formulations used in this experiment.

**Table 1:** Feed formulations used for the experiment

	Pre-starter diet			Starter diet		
	0% FRS	2.5% FRS	5% FRS	0% FRS	2.5% FRS	5% FRS
Ingredients, g/kg						
Wheat (11.2%)	613.26	597.88	583.86	539.88	525.11	508.24
Barley (10.6%)	100.00	100.00	100.00	200.00	200.00	200.00
Soybean meal (46.0%)	0.00	0.00	0.00	170.00	170.00	169.69
Digestible soy	79.08	69.35	59.32	10.63	0.77	0.00
Fermented rapeseed-seaweed meal	0.00	25.00	50.00	0.00	25.00	50.00
Potato protein	40.00	40.00	40.00	0.00	0.00	0.00
Fish meal (70%)	40.00	40.00	40.00	14.00	14.00	7.00
Whey protein	50.00	50.00	50.00	0.00	0.00	0.00
Soy bean oil	37.28	38.93	40.17	23.74	25.20	26.50
Limestone (Ca 38.5%)	0.00	0.00	0.00	5.71	5.57	5.66
Calcium formate	5.00	5.00	5.00	0.00	0.00	0.00
Calcium phosphate	9.12	8.68	8.23	8.83	8.38	8.29
Sodium chloride	3.83	3.70	3.58	4.98	4.85	4.89
Summer fruit	2.00	2.00	2.00	2.00	2.00	2.00
Tretracid liquid	5.82	5.83	5.84	5.60	5.61	5.75
Lysine HCl (98%)	5.00	4.00	2.50	5.00	4.00	2.50
Methionine DL (99%)	0.77	0.73	0.69	1.02	0.97	1.01
Threonine L (99%)	1.88	1.83	1.77	1.94	1.88	1.85
Valine (98%)	1.11	1.09	1.06	1.00	0.97	0.96
Tryptophan (99%)	0.65	0.65	0.65	0.37	0.37	0.36
Microbial phytase	0.15	0.15	0.15	0.15	0.15	0.15
Microbial xylanase, beta-glucanase	0.15	0.15	0.15	0.15	0.15	0.15
Vitamin E (50%)	0.03	0.03	0.30	0.10	0.01	0.01
Vitamin-mineral premix^a^	5.00	5.00	5.00	5.00	5.00	5.00
Calculated nutritive value, %						
Dry weight	88.50	88.50	88.60	87.50	87.50	87.60
Metabolizable energy, MJ	14.30	14.30	14.30	13.50	13.50	13.50
Crude protein	19.70	19.70	19.70	18.40	18.40	18.40
Crude fat	5.56	5.75	5.90	4.03	4.20	4.31
Crude fiber	2.20	2.38	2.57	3.11	3.29	3.51
Ashes	5.37	5.42	5.45	5.69	5.71	5.87
Starch, g	471.40	408.30	399.90	425.60	416.90	406.80
Lactose, g	36.50	36.50	36.50	0.00	0.00	0.00
Calcium	0.83	0.83	0.84	0.82	0.82	0.82
Total phosphorous	0.65	0.65	0.65	0.58	0.58	0.58
Digestible phosphorous	0.59	0.59	0.59	0.51	0.51	0.51
Sodium	0.23	0.23	0.23	0.22	0.22	0.22
Chlorine	0.57	0.56	0.56	0.51	0.50	0.50
Potassium	0.65	0.65	0.66	0.67	0.68	0.69
Lysine	1.46	1.46	1.46	1.28	1.28	1.28
Methionine	0.45	0.45	0.45	0.41	0.41	0.41
Met + Cys^b^	0.77	0.77	0.78	0.72	0.73	0.73
Threonine	0.92	0.92	0.92	0.81	0.81	0.81
Tryptophane	0.30	0.31	0.31	0.26	0.26	0.26
Valine	1.03	1.03	1.03	0.90	0.90	0.90
Isoleucine	0.78	0.78	0.78	0.70	0.69	0.69

^a^Provided the following per kilogram of feed: Vitamin A 13,000 IU; vitamin D_3_ 2000 IU; Vitamin E 165 mg; vitamin B_1_ 2.5 mg; vitamin B_2_ 7.0 mg; biotin 200 mcg; vitamin B_6_ 4 mg; vitamin B_12_ 50 mcg; vitamin K_3_ mg; Niacin 35 mg; folic acid 1.5 mg; Pantothenic acid 21.7 mg; vitamin C 100 mg; choline 0 mg; Fe 180 mg; Zn 150 mg; Cu 0 mg; Mn 55 mg; Se 0.4 mg; I 0.6 mg; Mg 0 mg. ^b^methionine + cysteine

These piglets were housed in the nursing units holding an average of 48 animals per pen. The FRS dietary treatment was repeated 5 times (1 repetition per experimental week and 1 pen representing a repetition) and the control was repeated 4 times. Piglets which experienced diarrhea or any other serious health conditions were removed from the experiment, treated elsewhere and counted as piglets that did not complete the experiment. Feed and fresh water were supplied ad libitum throughout the experiment. Piglet performance were recorded every week by pen. Performance indicators such as body weight, average daily feed intake (ADFI), average daily weight gain (ADG), feed conversion ratio (FCR) and the completion rate were calculated as previously outlined [5].

### Biological sample collection

A total of 10 piglets from each group (*n* = 5 in each of two experimental weeks) were randomly selected and euthanized 3 weeks after weaning. The animals were euthanized by stun gunning with a captive bolt immediately followed by de-bleeding at the farm slaughtering facilities under strict sanitary regulations. Whole blood samples and serum for clinical analysis, the digesta from the colon for microbiome analysis, and jejunum and colon tissues for histopathological analyses, were collected in that order immediately after slaughtering.

A blood sample from each piglet was deposited in a tube with the anti-coagulant EDTA and preserved on ice and taken to the laboratory, where it was stored at 2–8 °C until analysis. Another blood sample was collected in a tube without anticoagulant, and serum separated by centrifugation, which was then stored at − 20 °C until analysis. Gut tissues and colon contents were sampled after opening of the abdominal wall, and the stomach, small and large intestines were occluded at both ends and removed. Approximately 2 cm^3^ of colon content was collected from the apex of the ascending spiral of the colon with a sterile spatula and deposited in cryotubes with RNAlater™ (Sigma-Aldrich, Munich, Germany). The tubes with colon contents were kept at room temperature for less than 24 h, followed by cryopreservation in the laboratory. Tissue samples (approximate length of 2 cm) of the whole transection of the jejunum and colon were excised and carefully rinsed from gut contents by flushing with saline (0.9% NaCl). For each tissue a sterilized blade was used. The tissues were preserved in 10% formaldehyde and kept at room temperature for no longer than 24 h until further processing [20].

### Hematology, blood biochemistry and serum immunoglobulin analysis

Full blood counts (erythrocyte, hemoglobin, hematocrit, mean corpuscular volume, mean corpuscular hemoglobin, mean corpuscular hemoglobin concentration, red cell distribution width) and differential white blood cells count (platelets, leucocytes, lymphocytes, monocytes, neutrophils, eosinophils, basophils) were performed using a Sysmex XT 2000i analyzer (Sysmex Corporation, Kobe, Japan). Serum analysis followed the standardized quantification methods [5] to measure the concentrations of the following: alanine aminotransferase, glutamic pyruvic transaminase, aspartate aminotransferase, glutamic-oxaloacetic transaminase, lactate dehydrogenase, lysozyme, glucose, total protein, blood urea nitrogen, uric acid, phosphorous, total cholesterol, triglycerides, low density lipoprotein, high density lipoprotein and immunoglobulin G (IgG).

### Histological analysis of intestinal tissues

The histological analysis of mid-jejunal and colonic tissues was conducted by a commercial analytical laboratory (ALAB Weterynaria, Warsaw, Poland) according to previous procedures [5]. In short, tissue sections fixed in 10% formaldehyde were dehydrated by means of graded ethanol and xylene baths and embedded in paraffin wax, and 3–4 μm section were then stained with haematoxylin and eosin. Histopathological evaluations (at different lens magnifications) measured gut-associated lymphoid tissue (GALT), intraepithelial lymphocytes (IELs) and lymphatic infiltration of the stromal mucosa (stromal lymphocytes, SL) counts. For GALT, the numbers of lymphoid follicles per millimeter square were counted. For IEL scoring, the following scale was used: 0-normal (0–10 IELs/100 enterocytes), 1-low (10–15 IELs/100 enterocytes), 2-moderate (15–20 IELs/100 enterocytes; this level suggests chronic subclinical inflammation, where the intestinal-blood barrier may be damaged), 3-severe (> 20 IELs/100 enterocytes; this level indicates chronic inflammation with infiltration damaging the epithelium and intestinal-blood barrier). For SL, the visual scoring scale was: 0-normal (single lymphocytes in stromal connective tissues of villus and crypts), 1-low (increased number of lymphocytes, but no damage to the stroma structures), 2-moderate (abundant infiltration of lymphocytes in stroma, damaging blood vessel walls, connective tissue fiber, reducing visibility of stroma structures), 3-severe (lymphocyte infiltration completely disrupts and conceals the stroma). In a blinded fashion, 10 fields of view per piglet at 4× magnification were used for evaluation of GALT structures and numbers of lymphoid follicles. IEL and SL were evaluated at 40× magnification. The analysis used a standard light microscope Olympus BX41 and Cell Sens software (Olympus Corporation, Tokyo, Japan). The gut tissue samples which could not reach the requirements for histological analysis were discarded, resulting in *n* = 9, 8, 10 for the 0%, 2.5% and 5% FRS group, respectively.

### 16S rRNA gene amplicon sequencing of colon content

The collected colon contents were stored at − 60 °C prior to the analysis. Two types of 16S rRNA gene amplicon sequencing strategies were adopted to characterize the prokaryotic community: Illumina, NextSeq (Illumina, CA, USA) and MinION (Oxford Nanopore Technologies, Oxford, UK). The genomic DNA was extracted using Bead-Beat Micro AX Gravity Kit (A&A Biotechnology, Gdynia, Poland) according to the manufacturer’s instruction. DNA concentration and purity were measured using NanoDrop ND-1000 spectrophotometer (Saveen and Werner AB, Sweden).

Extracted DNA was diluted to 10 ng/μL prior to library preparation. The V3 hypervariable region of 16S rRNA gene was amplified and sequenced with Illumina technology as previously described [21]. The V1-V8 hypervariable region of 16S rRNA gene was amplified and sequenced with ONT using the following primers: ONT_27Fa: GTCTCGTGGG CTCGGAGATG TGTATATAGA TCGCAGAGTT TGATYMTGGCTCAG; ONT_27Fb: GTCTCGTGGG CTCGGAGATG TGTATATAGA TCGCAGAGTT TGATCCTGGCTTAG and ONT_1540_R: GTCTCGTGGG CTCGGAGATG TGTATACTCT CTATTACGGY TACCTTGTTACGACT. Custom designed barcoding system was developed to tag encode up to 96 samples during the second round of PCR, and the PCR primer sequence is given in Table S1 (Additional file). The PCR1 reaction mix contained 5 μl of PCRBIO buffer and 0.25 μL PCRBIO HiFi polymerase (PCR Biosystems Ltd., London, United Kingdom), 1 μL of primers mix (5 μmol/L of ONT_27Fa and ONT_27Fb, and 10 μmol/L of ONT_1540_R, see above), 5 μL of genomic DNA (~ 10 ng/μL) and nuclease-free water to a total value of 25 μL. The PCR thermal conditions were as follows: denaturation at 95 °C for 5 min; 33 cycles of 95 °C for 20 s, 55 °C for 20 s and 72 °C for 45 s; followed by final elongation at 72 °C for 4 mins.

PCR products were verified by agarose gel electrophoresis and then subjected for barcoding (PCR2). The PCR2 mix composed of 5 μL PCRBIO buffer, 0.25 μL PCRBIO HiFi polymerase (PCR Biosystems Ltd., London, United Kingdom), 2 μL of barcode primers (5 μmol/L), 1 μL of PCR1 template and DEPC water up to 25 μL. The PCR2 thermal conditions were as follows: denaturation at 95 °C for 2 mins; 13 cycles of 95 °C for 20 s, 55 °C for 20 s, 72 °C for 40 s; final elongation at 72 °C for 4 mins. The final PCR products were purified using AMPure XP beads (Beckman Coulter Genomic, CA, USA) and pooled in equimolar concentrations. The pooled barcoded amplicons were subjected to 1D genomic DNA by ligation protocol (SQK-LSK109) to complete library preparation for MinION sequencing. Approximate 0.2 μg of amplicons were used for the initial step of end-prep. And 40 ng of prepared amplicon library was loaded on a R9.4.1 flow cell.

### Sequencing data analysis

The raw Illumina pair-ended reads were merged and trimmed using fastq_mergepairs and fastq_filter scripts implemented in the USEARCH pipeline as outlined [21]. The chimeric reads was removed and the zero radius Operational Taxonomic Units (zOTUs) conducted using the UNOISE3 algorithm [22]. The Greengenes (13.8) 16S rRNA gene collection was used as a reference database for taxonomy assignment [23].

Data generated by MinION were collected using MinKnow software v19.06.8 (https://nanoporetech.com). The Guppy v3.2.2 basecalling toolkit was used to base call raw fast5 to fastq (https://nanoporetech.com). Porechop v0.2.2 was used for adapter trimming and sample demultiplexing (https://github.com/rrwick/Porechop). The Porechop adapter list was (adapters.py) edited accordingly and is given in Table S1 (Additional file). Sequences containing quality scores (fastq files) were quality corrected using NanoFilt (q ≥ 10; read length > 1Kb). Taxonomy assignment of quality corrected reads against Greengenes (13.8) database was conducted using uclast method implemented in parallel_assign_taxonomy_uclust.py (QIIME v1.9.1). The uclust settings were tuned on mock communities (−-similarity 0.8; min_consensus_fraction 0.51) assuring annotations to the lowest taxonomic level with no false positive annotations. The settings allowed it to treat individual amplicon sequence variants as individual “seeds”. Reads classified to at least phylum level were subjected for further analysis.

### Statistics

All the statistical analysis of phenotypic data was performed with R (v3.6.2). The piglet production performance was evaluated using linear mixed model to control the confounding variables like pen difference as previously described [5]. Orthogonal polynomial contrast was used to appreciate the effect on increasing dose of the FRS (0%, 2.5%, 5%). The performance analysis took 28 days of life as assessment starting point and the last 2 weeks in the pre-starter (42 and 49 days of life) and starter diet period (77 and 85 days of life) as the end point. The blood hematology and biochemistry data were analyzed by R package compareGroups [24] (v4.0) using “comparaGroups” command and the descriptive table was generated by “createTable” command. In R package compareGroups, the significant differences among groups were determined by ANOVA and Tukey’s procedure for post hoc tests. Wilcoxon rank-sum test was used to evaluate the histological difference between groups.

For microbiome analysis, QIIME 2 [25] (v2018.11) and R packages ggplot2, vegan, corrplot, Rhea, rstatix and vennDiagram were used. Three samples were removed due to inadequate library size (< 1,000 counts), resulting in *n* = 9, 8, 10 for 0%, 2.5% and 5% FRS group, respectively. For both sequencing strategies, all the samples were summarized at the L7 levels (species) and rarefied to the same sequencing depth (11,000 reads/sample) for alpha and beta diversity calculations. Rarefaction on the zOTU table (Illumina data) was adopted as comparison for rarefaction on the species-level summarized table. The sequencing depth (11,000 reads/sample) was assessed with the good’s coverage index and it ranged between 99.7% to 100% for all samples. Principal coordinate analysis (PCoA) plots were generated using binary Jaccard and Bray Curtis distance metrics, and PERMANOVA was performed to determine differences between groups and *P* values were adjusted by Benjamini-Hochberg correction. ANCOM [26] was adopted to identify differentially abundant taxa summarized at L7 level. For identified taxa by ANCOM, Wilcoxon rank-sum test was adopted for pairwise comparison. Phenotypic data were integrated with species-level bacterial abundances by Pearson’s correlation analysis using R package Rhea [27]. Rare microbial features were removed with a cutoff of mean relative abundance > 0.1% and minimal presence among 30% of samples. Zeros of microbial feature abundance were regarded as NA and excluded in the correlation analysis to avoid the bias induced by extreme values. Centered log-ratio transformation was conducted in both the microbial relative abundance and phenotypic data.

## Results

### Piglet performance, blood hematology, blood biochemistry and systemic immunoglobulin

The performance data in the last 2 weeks of the pre-starter and starter diet period were taken to assess changes under different feeding regimes. Piglets fed with 2.5% FRS showed numerically increased final body weight (85 days of age) in comparison with those on the basal diet. However, orthogonal polynomial contrast indicated no significant differences in body weight, ADFI, ADG, FCR between the three groups (Table 2). The completion rate for piglets in the experiment (i.e., not dead or removed due to the need for antibiotics treatment) did not differ between the treatment groups. In the sub-group of piglets euthanized 3 weeks after weaning, we found no statistical differences in blood hematology, blood chemistry and systemic immunoglobulin parameters between treatment groups, except for the levels of blood urea nitrogen (BUN) and mean corpuscular volume (Table 3). Relative to piglets fed without FRS inclusion, 2.5% FRS reduced plasma concentrations of BUN, but increased mean corpuscular volume. The piglets in 5% FRS group showed a similar tendency, but only with significantly declined BUN concentrations.

**Table 2:** Performance of piglets subjected to three dietary regimens

Parameters	0% FRS	2.5% FRS	5% FRS	SEM	*P* value		
					TG	TG	
						L	Q
Weaning weight, kg (d 28)	6.07 ± 0.33	6.24 ± 0.34	5.99 ± 0.66				
Body weight at age of interest, kg							
42	6.77	7.00	6.55	0.406	0.579	0.890	0.428
49	8.24	8.75	8.41	0.418	0.563	0.746	0.314
77	20.5	21.6	20.2	1.18	0.537	0.842	0.341
85	23.2	25.1	23.7	1.82	0.467	0.823	0.269
ADFI, kg/d							
d 28–42	0.162	0.183	0.166	0.015	0.437	0.830	0.231
d 28–49	0.233	0.232	0.230	0.020	0.985	0.900	0.967
d 28–85	0.516	0.572	0.546	0.037	0.418	0.477	0.236
d 50–77	0.626	0.661	0.632	0.031	0.531	0.879	0.299
d 50–85	0.671	0.763	0.719	0.058	0.365	0.460	0.199
ADG, kg/d							
d 28–42	0.049	0.063	0.049	0.025	0.835	0.993	0.574
d 28–49	0.103	0.122	0.118	0.017	0.681	0.506	0.499
d 28–85	0.306	0.339	0.316	0.034	0.584	0.790	0.343
d 50–77	0.439	0.456	0.420	0.037	0.623	0.702	0.476
d 50–85	0.423	0.467	0.431	0.045	0.486	0.993	0.574
FCR							
d 28–42	6.78	7.42	7.13	3.12	0.938	0.878	0.740
d 28–49	2.37	2.12	2.09	0.311	0.681	0.486	0.683
d 28–85	1.80	1.68	1.76	0.147	0.584	0.834	0.515
d 50–77	1.44	1.45	1.55	0.084	0.485	0.365	0.597
d 50–85	6.78	7.42	7.13	3.12	0.938	0.878	0.740
Completion rate, %							
d 28–42	95.2	96.9	96.1	2.10	0.773	0.74	0.504
d 28–49	94.1	95.1	94.3	2.29	0.847	0.921	0.601
d 28–85	94.8	94.4	89.1	3.60	0.472	0.328	0.564

*ADFI* Average daily feed intake; *ADG* Average daily gain; *FCR* Feed conversion ratio; *TG* Treatment group; *L* Linear effect; *Q* Quadratic effect; The statistical significance between different dosage group is based on the result of orthogonal contrasts

**Table 3:** Blood hematology and biochemistry analysis of piglets under the three feeding regimens. Respectively *n* = 10, 10, 10 for the feeding regimens with 0%, 2.5% and 5% added lacto-fermented rapeseed-seaweed blend (FRS), respectively

	0% FRS (*n* = 10)	2.5% FRS (*n* = 10)	5% FRS (*n* = 10)	*P*.overall	*P*.0% FRS vs. 2.5% FRS	*P*.0% FRS vs. 5% FRS	*P*.2.5% FRS vs. 5% FRS
Erythrocyte count, 10^12^cells/L	6.12 [5.77;6.47]	5.75 [5.43;6.07]	5.87 [5.48;6.26]	0.25	0.23	0.50	0.85
Hemoglobina, g/dL	10.06 [9.53;10.59]	10.07 [9.37;10.77]	9.81 [9.16;10.46]	0.76	1.00	0.80	0.79
Hematocrit, %	33.37 [31.51;35.23]	33.74 [30.92;36.56]	32.21 [29.92;34.50]	0.56	0.97	0.71	0.56
Mean corpuscular volume, fL	54.65 [52.42;56.88]	58.73 [55.51;61.95]	54.98 [52.87;57.09]	0.03	0.04	0.98	0.07
Mean corpuscular hemoglobin, pg/cell	16.49 [15.83;17.15]	17.55 [16.76;18.34]	16.77 [16.04;17.50]	0.07	0.07	0.81	0.22
Mean corpuscular hemoglobin concentration, g/dL	30.20 [29.19;31.21]	29.93 [28.76;31.10]	30.49 [29.72;31.26]	0.67	0.90	0.89	0.65
Red cell distribution width, %	24.00 [22.30;25.70]	22.37 [19.66;25.08]	24.25 [21.95;26.55]	0.37	0.49	0.98	0.40
Platelets, 10^11^cells/L	3.74 [3.11;4.38]	3.44 [2.61;4.28]	3.35 [2.48;4.21]	0.71	0.81	0.70	0.98
Leucocyte, 10^10^cells/L	3.64 [2.67;4.61]	2.93 [2.31;3.54]	2.97 [2.40;3.54]	0.24	0.29	0.33	0.99
Neutrophile, %	55.15 [43.11;67.19]	47.54 [38.74;56.34]	56.39 [47.12;65.66]	0.33	0.46	0.98	0.36
Limphocytes, %	40.73 [28.77;52.69]	47.14 [37.79;56.49]	38.05 [28.54;47.56]	0.37	0.59	0.91	0.35
Monocytes, %	3.37 [1.59;5.15]	4.21 [2.68;5.74]	4.57 [2.04;7.10]	0.62	0.78	0.60	0.95
Eosinophile, %	0.21 [<0.01;0.42]	0.73 [0.09;1.37]	0.66 [0.01;1.31]	0.27	0.29	0.39	0.98
Basophile, %	0.54 [0.08;1.00]	0.38 [0.11;0.65]	0.33 [0.09;0.57]	0.59	0.73	0.59	0.97
Alanine aminotransferase, units/L	54.80 [40.63;68.97]	45.70 [39.21;52.19]	48.90 [37.41;60.39]	0.43	0.41	0.68	0.89
Aspartate aminotransferase, units/L	120.50 [40.71;200.29]	69.40 [54.69;84.11]	85.40 [56.14;114.66]	0.26	0.25	0.51	0.87
Lactate dehydrogenase, units/L	1072.20 [690.90;1453.50]	960.30 [836.86;1083.74]	969.10 [702.71;1235.49]	0.78	0.80	0.82	1.00
Glucose, mg/dL	102.10 [91.99;112.21]	110.00 [95.14;124.86]	105.90 [86.28;125.52]	0.72	0.69	0.92	0.90
Total protein, g/dL	4.41 [3.86;4.96]	4.68 [4.30;5.06]	4.87 [4.15;5.59]	0.44	0.73	0.41	0.85
Blood urea nitrogen, mg/dL	19.50 [16.04;22.96]	10.00 [5.29;14.71]	13.20 [9.87;16.53]	<0.01	<0.01	0.04	0.40
Phosphorous, mg/dL	9.65 [7.82;11.48]	9.52 [8.38;10.66]	8.80 [7.33;10.27]	0.63	0.99	0.64	0.73
Cholesterol, mg/dL	73.00 [57.36;88.64]	70.00 [59.74;80.26]	62.60 [53.55;71.65]	0.37	0.92	0.36	0.59
Triglycerides, mg/dL	44.00 [25.70;62.30]	49.30 [35.66;62.94]	41.50 [33.51;49.49]	0.66	0.82	0.96	0.65
Cholesterol LDL^a^, mg/dL	39.94 [30.50;49.38]	38.39 [30.86;45.92]	33.71 [27.46;39.96]	0.43	0.95	0.42	0.61
Cholesterol HDL^b^, mg/dL	33.99 [26.12;41.86]	34.33 [28.81;39.85]	29.48 [24.02;34.94]	0.41	1.00	0.50	0.45
IgG, μg/mL	2625.95 [2075.63;3176.26]	2163.86 [1945.63;2382.08]	2559.73 [2110.51;3008.95]	0.20	0.22	0.97	0.32
Lysozyme, pmol/mL	168.09 [119.95;216.22]	154.15 [121.86;186.44]	156.09 [119.40;192.78]	0.83	0.84	0.88	1.00

^a^High-density lipoprotein

^b^Low-density lipoprotein

### High level of accordance between the short and long read amplicon sequencing strategies

**Fig. 1:**
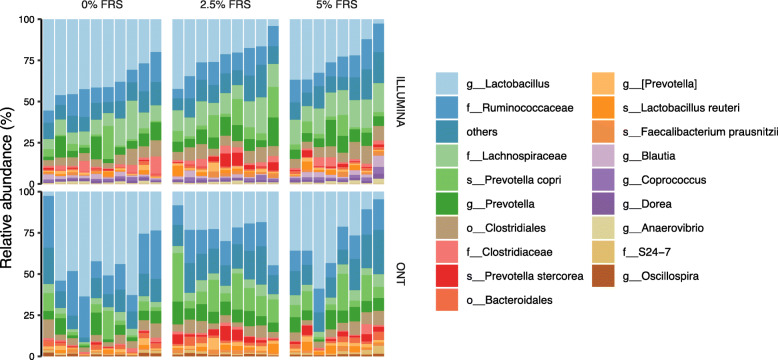
The colon microbiota profiles of weaner piglets under FRS feeding regimens. The microbial features are collapsed at the lowest identified taxonomic assignments. Respectively *n* = 9, 8, 10 for feeding regimens with 0%, 2.5% and 5% added lacto-fermented rapeseed-seaweed blend (FRS)

Two different sequencing strategies were applied: Illumina NextSeq-based amplicon sequencing of the 16S rRNA gene V3 variable region (Illumina V3) and ONT based sequencing of V1-V8 variable regions (ONT V1-V8). Out of 99 unique taxonomic groups found by the two methods, 78 were shared (Additional file 1: Fig. S1A). The accordance was further improved when abundance threshold was adjusted (Additional file 1: Fig. S1B-D). The taxonomic groups with relative abundance above 3% were identical in the two methods (Additional file 1: Fig. S1E) and the overlapped detections overall showed good positive correlation in between (Additional file 1: Fig. S1F). Both methods revealed that the most dominant bacterial groups belonged to genus *Lactobacillus* and families: *Ruminococcae* and *Lachnospiraceae* independent of treatment (Fig. 1).

### Dietary inclusion of FRS induced distinct shifts in the colon microbiota composition

**Fig. 2:**
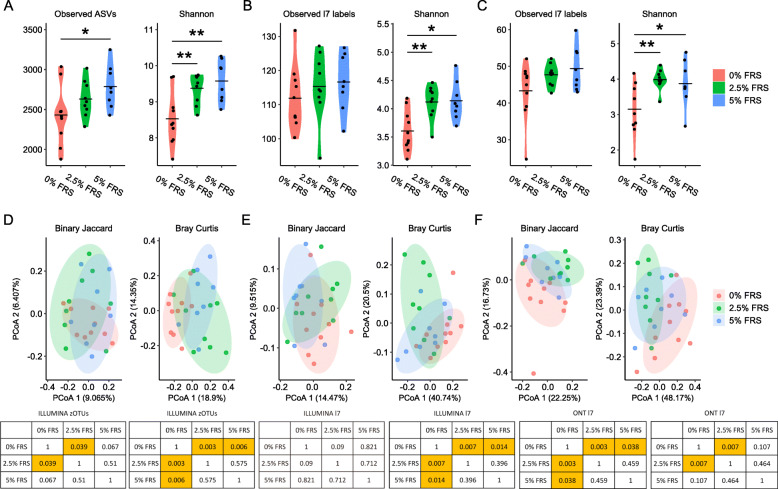
Dietary inclusion of FRS induced distinct shifts in the colon microbiota composition. Observed zOTUs and Shannon diversity based on rarefied zOTU table with Illumina sequencing on V3 region (Illumina V3) (**a**); Observed features and Shannon diversity based on species-level summarized table by Illumina V3 (**b**) and Oxford Nanopore sequencing on V1-V8 region (ONT V1-V8) (**c**). The mean value for each group is marked as a bold line respectively. PCoA plots of binary Jaccard and Bray Curtis distance metrics based on the rarefied ASV table of Illumina V3 (**d**), species-level summarized table of Illumina V3 (**e**) and species-level summarized table of ONT V1-V8 (**f**). The ellipses suggest the respective 80% confidential area following multivariate t-distribution. Respectively *n* = 9, 8, 10 for feeding regimens with 0%, 2.5% and 5% added lacto-fermented rapeseed-seaweed blend (FRS). For pairwise Wilcoxon rank-sum tests on alpha diversity metrics, the labels of *, ** represent *P* < 0.05, < 0.01 respectively. For PERMANOVA tests, *P* values below 0.05 are heighted in yellow

Both sequencing approaches revealed alterations in gut microbial diversity in piglets under different feeding regimens. FRS inclusion resulted in increased Shannon diversity and observed features. The effect was consistent, when the analysis was performed based on the zOTU table (Fig. 2a), and the summarized species-level table (Fig. 2b) from Illumina data and ONT data (Fig. 2c). Increasing the FRS inclusion from 2.5% to 5% did not lead to significant changes of alpha diversity between the two groups (Fig. 2a–c). Beta diversity analysis on binary Jaccard (qualitative) and Bray Curtis dissimilarity metrics (quantitative) indicated that introduction of FRS in the feed influenced the colon microbiota composition of weaner piglets. These changes were more pronounced in the 2.5% FRS group relative to the 5% FRS (Fig. 2d-f).

### Dietary inclusion of 2.5% FRS increased the *Prevotella stercorea* and *Mitsuokella* abundance in colon

**Fig. 3:**
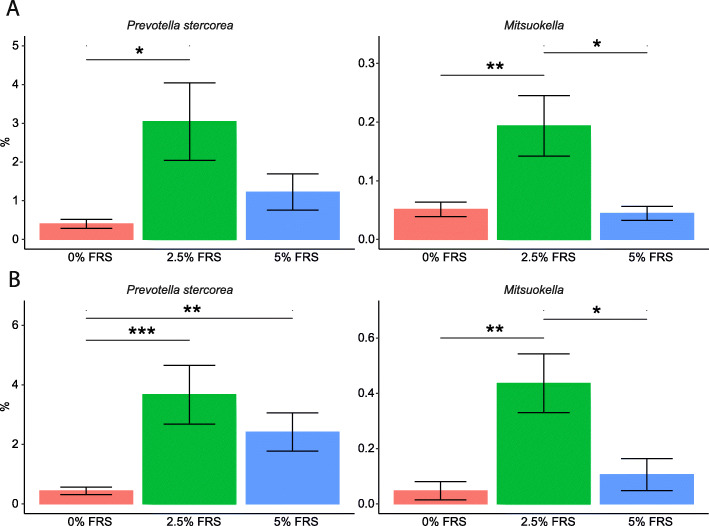
Dietary inclusion of 2.5% FRS increased the *Prevotella stercorea* and *Mitsuokella* abundance in colon. The *Prevotella stercorea* and *Mitsuokella* spp. abundances determined by Illumina sequencing on V3 region of 16S rRNA gene (**a**) and Oxford Nanopore sequencing on V1-V8 region of 16S rRNA gene (**b**). Data in the bar plot was presented as mean value and SEM error bar. Respectively *n* = 9, 8, 10 for feeding regimens with 0%, 2.5% and 5% added lacto-fermented rapeseed-seaweed blend (FRS). The labels of *, **, *** represent *P* < 0.05, < 0.01, < 0.005 respectively

**Fig. 4:**
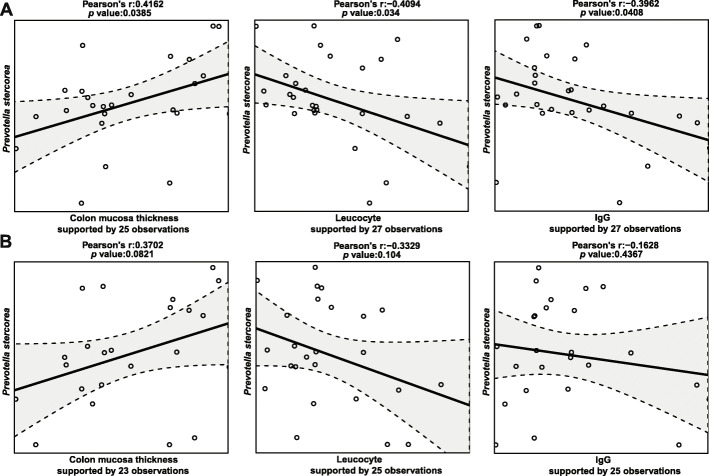
Colon *Prevotella stercorea* abundance was associated with colon mucosa thickness, serum leucocyte and IgG concentrations. The correlations pairs were statistically significant using the taxa abundance from V3 region 16S rRNA gene sequencing (**a**), similar trends yet not significant were found using the taxa abundance from V1-V8 region 16S rRNA gene sequencing (**b**)

The relative abundances of *Prevotella stercorea* and *Mitsuokella* spp. were increased in the 2.5% FRS-feeding group compared to the none-FRS diet and for most comparisons also in 5% FRS (Fig. 3a–d). The relative abundance of *Prevotella stercorea* (Illumina V3) was shown to be positively correlated with the colon mucosa thickness and negatively correlated to the blood leucocytes counts and serum IgG concentrations (Fig. 4a), while this observation was only near-significant using ONT (Fig. 4b).

There were 83 significant correlation pairs between bacteria relative abundance by Illumina V3 sequencing and phenotypic indicators, while ONT resulted in 114 significant pairs. Although two methods gave similar associations and trends for the overlapped pairs, only one taxon, *Faecalibacterium prausnitzii* showed identical accordance in correlations to the phenotypic data. The relative abundance of *F. prausnitzii* was positively correlated with colon mucosa thickness but negatively associated with the serum concentrations of aspartate aminotransferase, lactate dehydrogenase and IgG (Additional file 2: Fig. S2).

### Gastrointestinal histological analysis of the weaned piglets

**Fig. 5:**
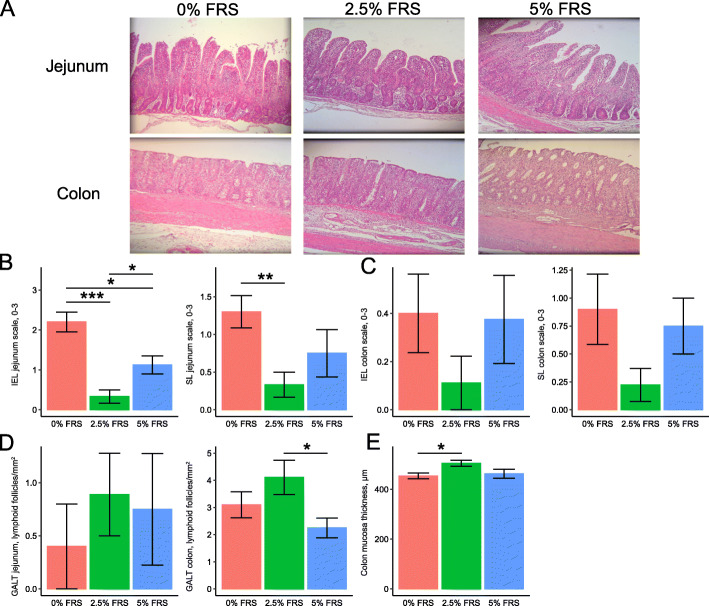
Gastrointestinal histological analysis of the weaned piglets. Histopathological micrograph of jejunum and colon under 10× magnification (**a**), gastrointestinal inflammation measured by the number of lymphocytes infiltrated at the epithelium (IEL) and stroma (SL) of jejunum (**b**) and colon (**c**), the numbers of lymphoid follicles per squared millimeter in jejunum and colon (**d**) and colon mucosa thickness (**e**). Respectively *n* = 9, 8, 10 for feeding regimens with 0%, 2.5% and 5% added lacto-fermented rapeseed-seaweed blend (FRS). The labels of *, **, *** represent *P* < 0.05, < 0.01, < 0.005 respectively

The morphological characteristics of intestinal tissues obtained from piglets in the different treatment groups are shown in Fig. 5a. All animals fed 0%, 2.5% and 5% FRS presented normal ranges for heights and structures of villi and intestinal crypts. The continuity and height of the jejunal and colonic epithelium were more pronounced in both FRS groups compared to the piglets on the basal feed with no added FRS. In the jejunal epithelium and stroma, the piglets fed with FRS had reduced IEL and SL infiltration compared to 0% FRS group, with 2.5% FRS showing the best effect (Fig. 5b). We found the similar tendency of alleviated focal inflammation in the colon tissues of 2.5% FRS, but with no significant difference between treatment groups (Fig. 5c). Diffuse lymphoid follicles at the base of the mucosa were visible with normal size and structure in jejunum and colon. No clear stimulation of lymphoid follicles was observed in all gut tissues. Neither did FRS inclusion result in the aggregation of jejunal and colonic lymphoid follicles (Fig. 5d). Histological evaluation did not show damaged intestinal epithelial barrier in any group but the mucous membrane was higher with deeper intestinal crypts in the 2.5% FRS relative to 0% FRS (Fig. 5e).

## Discussion

In modern pig production, the weaning of piglets is usually conducted at an early age, with physiological stress from changes in diet, environment and social groups. Hence, many weaned piglets experience intestinal and immune dysfunction, elevated risk of infection with enteric pathogens, diarrhea, and lowered weight gain due to reduced feed intake and poorer utilization of ingested nutrients [28]. Reduced weight gain and high mortality rate among the weaned piglets are undesirable in production. Preventive measures, such as use of in-feed antibiotics, have been banned by the European Union (EU) back in 2006, while the commonly used zinc oxide will be banned in 2022. Therefore, there is an urgent need for alternative strategies. We have shown that FRS feed increased the jejunal villus development of weaned piglets, stimulated colon mucosal development and reduced signs of intestinal inflammation [5]. Since FRS was demonstrated to be effective without in-feed zinc oxide, in the present study, we further investigated the dose-dependent influence of FRS on gut microbiome composition and its plausible link with phenotypic indicators.

To study the gut microbiome composition, we have adopted two sequencing strategies on different regions of microbial 16S rRNA gene. The short-read 16S rRNA gene amplicon sequencing by Illumina platform was compared with the near-full length 16S rRNA gene sequencing method by ONT. The two strategies showed satisfying accordance and allowed to draw the same overall conclusions including the taxonomic detections at the species level, which is challenging even when different hypervariable regions of 16S rRNA gene are profiled with the same sequencing platform [29–31].

Data generated with both sequencing strategies confirmed significant changes of gut microbiota composition in response to dietary FRS supplementation. This effect was more pronounced in the 2.5% FRS group relative to the 5% FRS. The colon microbiota of piglets under FRS feeding regimen had increased Shannon diversity, suggesting a more diverse and uniformly distributed microbial community than the piglets fed the basal diet. High microbial diversity is generally desirable, as it has been demonstrated to exclude pathogenic microbes, improve immune response and reduce necrotizing enterocolitis and post-weaning diarrhea incidences [32–34]. FRS supplementation led to increased relative abundance of *Prevotella stercorea* and *Mitsuokella* spp., especially pronounced in the 2.5% FRS group. *Prevotella* is known to be the major contributor to the microbiome of post-weaned piglets given the ability to degrade plant fibers in the solid diet. The species *P. stercorea* has previously been described as a member of the healthy pig gut microbiome [35] and also a potent producer of SCFAs [36, 37], which maintain the intestinal barrier function through providing energy resources and immunoregulatory regulation [38–40]. Our data indicated that the abundance of *P. stercorea* correlated positively with colon mucosa thickness, which is not surprising, since *Prevotella* spp. are recognized colonizers of the mucosal sites [41]. It is also reported that complex hemicelluloses and cellulose most likely enhances the mucosal abundance of *P. stercorea* [42, 43]. The negative correlation of *P. stercorea* with the serum levels of leucocytes and IgG could suggest that increased abundance of *P. stercorea* on the more fibrous FRS supplemented diet stimulated the gut barrier and immune function, reducing the risk of systemic inflammation.

*Faecalibacterium prausnitzii* is one of the main butyrate producers found in the gastrointestinal tract [44]. Butyrate plays a vital role in gut physiology and gut health, serving as a main energy source for the colonocytes and a protector against inflammatory disease and colorectal cancer [45, 46]. Many studies have linked the reduced abundance of *F. prausnitzii* with different intestinal disorders, hence it has been proposed that *F. prausnitzii* is a potential biomarker of gut health [47]. Our data indicated that *F. prausnitzii* abundance in colon correlated positively with colon mucosa thickness and negatively with serum levels of two enzymes released from the liver i.e., aspartate aminotransferase and lactate dehydrogenase, and IgG. Increased concentration of the hepatic enzymes in blood is a sign of liver malfunction while IgG is a systemic indicator of host inflammation. Our findings are in line with studies demonstrating the ability of *F. prausnitzii* to reduce inflammation and improve the liver function in murine models [48, 49] and human trials [50].

Although the microbiota data suggested there was no distinct impact with regards to the inclusion levels of FRS (2.5% versus 5%), it is important to note that we found significantly alleviated lymphocyte invasion in jejunum and enhanced colon mucosa barrier function solely among piglets receiving 2.5% FRS. Meanwhile, inclusion of 5% FRS did not lead to further improvements of production performance (ADG, FCR, completion rate). Possibly, if excessive FRS are added, the piglets are exposed to more bioactive components from either the rapeseed or seaweed. Even though they are beneficial in low amounts, excessive intake can increase the physiological stress in the weaning period and become counterproductive to FCR. Young animals are more sensitive to the anti-nutritional factors like glucosinolates [51] than adult animals, and thereby our results indicate that the inclusion of 2.5% FRS is a superior choice than 5% FRS.

## Conclusion

Our study demonstrates that inclusion of 2.5% FRS in feed alleviated signs of lymphocyte invasion in jejunum and improved the colon mucosa barrier with reshaped microbiota community. 2.5% FRS supplementation led to increased microbial diversity in colon and elevated the abundances of *Prevotella stercorea* and *Mitsuokella* spp.

## Supplementary Information

**Additional file 1: Fig. S1.** Consistent results from short- and long-read amplicon sequencing of 16S rRNA gene. Venn plots of taxonomic features (collapsed at species level) from Illumina and ONT sequencing with mean relative abundance cut-off of 0% (A), 0.1% (B), 1% (C), 2% (D), 3% (E), and the Pearson’s correlation heatmap between the shared taxa (F). The annotations of Illumina species on rarefied ASVs, Illumina rarefied species and ONT indicate the captured labels at lowest taxonomic level using the rarefied Illumina ASV table, Illumina and ONT species-level summarized tables, respectively.

**Additional file 2: Fig. S2.** Correlation heatmap between the phenotypic parameters and colon microbiota composition. The color depth and size of the point represent the coefficient and *P* value respectively. Statistically significant pairs with *P* < 0.05 are marked with *. ILLUMINA, V3 region sequencing of 16S rRNA gene; ONT, V1-V8 region sequencing of 16S rRNA gene.

**Additional file 3: Table S1.** The PCR primer sequences for ONT V1-V8 region 16S rRNA gene sequencing.

## Data Availability

Raw sequencing data are available from the corresponding author on reasonable request.

## Abbreviations

ADFI: Average daily feed intake
ADG: Average daily weight gain
ANCOM: Analysis of compositions of microbiomes
BUN: Blood urea nitrogen
FRS: Rapeseed-seaweed blend fermented by lactobacilli
FCR: Feed conversion ratio
GALT: Gut-associated lymphoid tissue
IgG: Immunoglobulin G
IEL: Intraepithelial lymphocyte
SL: Stromal lymphocyte
ONT: Oxford nanopore technologies
PCR: Polymerase chain reaction
PCoA: Principal coordinate analysis
PERMANOVA: Permutational multivariate analysis of variance
QIIME: Quantitative insights into microbial ecology
SCFA: Short-chain fatty acids
zOTU: Zero radius operational taxonomic units
