# Supplementary figures and images for: Supplementation of a lacto-fermented rapeseed-seaweed blend promotes gut microbial- and gut immune-modulation in weaner piglets
Source: J Anim Sci Biotechnol. 2021 Jul 20;12:85. doi: 10.1186/s40104-021-00601-2 (PMC8290543; doi:10.1186/s40104-021-00601-2)

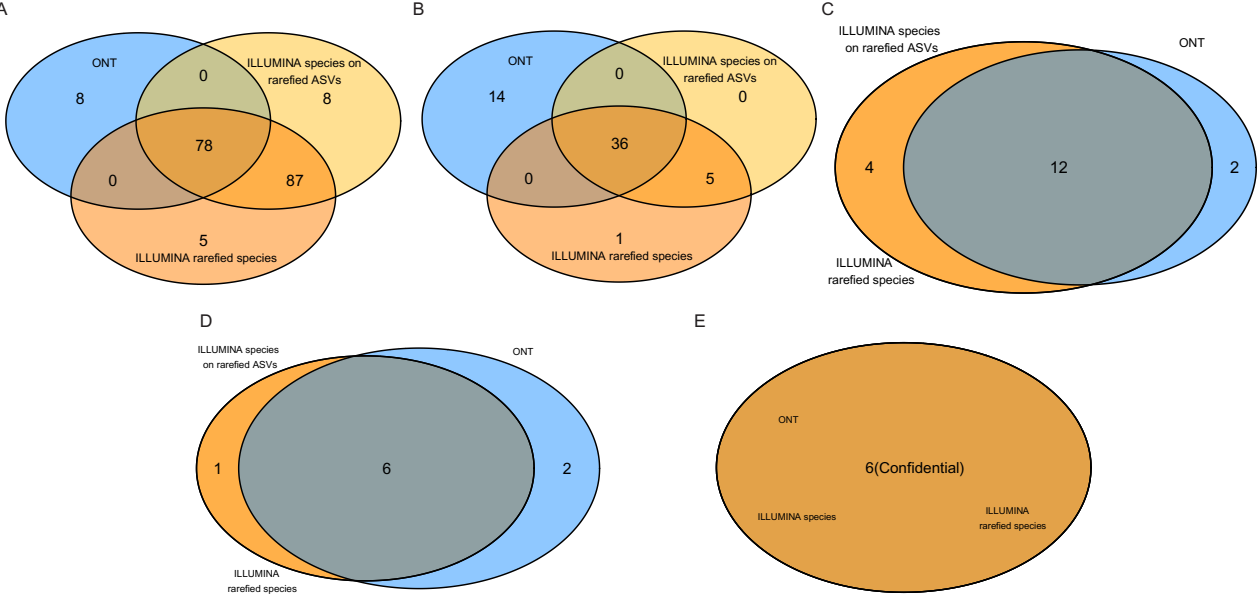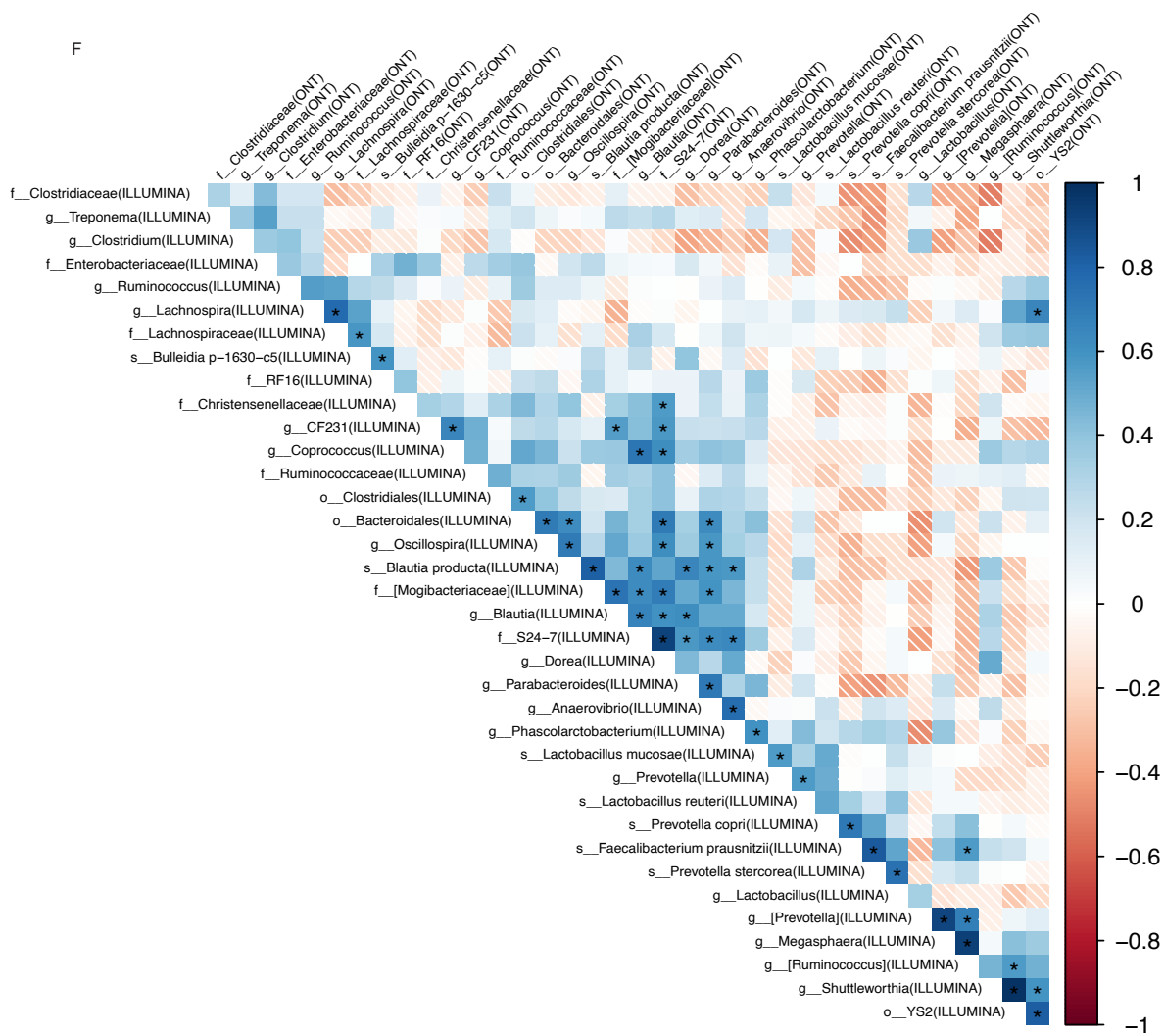

Supplement: Supplementary file 1 — Additional file 1: Fig. S1. Consistent results from short- and long-read amplicon sequencing of 16S rRNA gene. Venn plots of taxonomic features (collapsed at species level) from Illumina and ONT sequencing with mean relative abundance cut-off of 0% (A), 0.1% (B), 1% (C), 2% (D), 3% (E), and the Pearson’s correlation heatmap between the shared taxa (F). The annotations of Illumina species on rarefied ASVs, Illumina rarefied species and ONT indicate the captured labels at lowest taxonomic level using the rarefied Illumina ASV table, Illumina and ONT species-level summarized tables, respectively. [file 40104_2021_601_MOESM1_ESM.pdf]

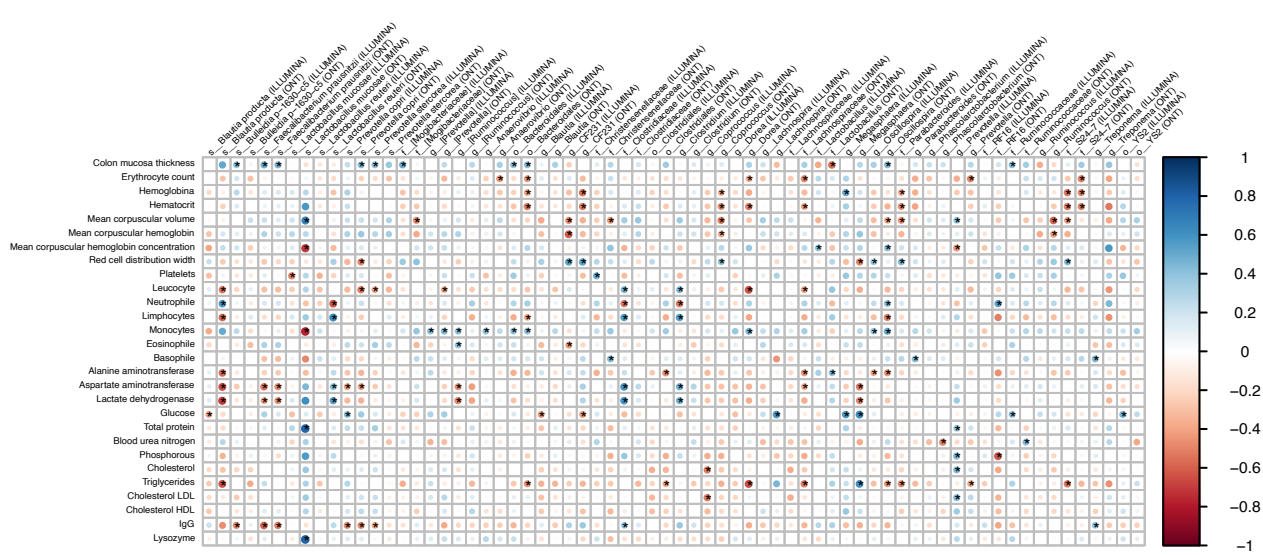

Supplement: Supplementary file 2 — Additional file 2: Fig. S2. Correlation heatmap between the phenotypic parameters and colon microbiota composition. The color depth and size of the point represent the coefficient and P value respectively. Statistically significant pairs with P < 0.05 are marked with *. ILLUMINA, V3 region sequencing of 16S rRNA gene; ONT, V1-V8 region sequencing of 16S rRNA gene. [file 40104_2021_601_MOESM2_ESM.pdf]
